# Identification of Pharmacological Autophagy Regulators of Active Ulcerative Colitis

**DOI:** 10.3389/fphar.2021.769718

**Published:** 2021-12-01

**Authors:** Peishan Qiu, Lan Liu, Jun Fang, Meng Zhang, Haizhou Wang, Yanan Peng, Min Chen, Jing Liu, Fan Wang, Qiu Zhao

**Affiliations:** ^1^ Department of Gastroenterology, Zhongnan Hospital of Wuhan University, Wuhan, China; ^2^ Hubei Clinical Center & Key Lab of Intestinal & Colorectal Diseases, Wuhan, China

**Keywords:** autophagy, ulcerative colitis, WGCNA, biomarkers, pharmacology

## Abstract

**Background:** Ulcerative colitis (UC) is a chronic recurrent disease of unknown etiology. Recently, it has been reported that autophagy-related gene polymorphism is closely associated with increased risk of UC, and the therapeutic effect of some UC drugs is mediated by regulating autophagy pathways. This study aims to identify pivotal autophagy-related regulators in UC pathogenesis and provide novel molecular targets for the treatment of active UC.

**Methods:** Gene expression profiles and clinical information of active UC patients were obtained from GEO databases. CIBERSORT was adopted to evaluate the immune cell infiltration. We used weighted gene co-expression network analysis (WGCNA) and differential expression analysis to identify the pivotal modules and genes associated with active UC. Subsequently, we conducted validation in the validation set and explored its relationship with commonly used UC therapeutics.

**Results:** 36 healthy controls and 46 active UC patients have been obtained from the training set of GSE53306, GSE87466, and GSE134025. There were 423 differentially expressed genes (DEGs) found, which dramatically enriched in autophagy-related pathways. And more infiltration of mast cells, activated T cells, dendritic cells, and M1 macrophages were observed in the intestinal mucosa of active UC, while more infiltration of resting immune cells and M2 macrophages in healthy controls. WGCNA indicated that the turquoise and blue modules were the critical modules. CASP1, SERPINA1, and CCL2 have been identified as the hub autophagy-related genes of active UC, after combining DEGs and 232 autophagy-related genes from HADb with the genes of turquoise and blue modules, respectively. We further verified that CASP1, SERPINA1, and CCL2 were positively associated with active UC and served as an autophagy-related biomarker for active UC. Moreover, increased SERPINA1 in the involved intestinal mucosa was reduced in patients with active UC who responded to golimumab or glucocorticoid therapy. But, neither CASP1, SERPINA1, and CCL2 were changed by treatment of 5-aminosalicylic acid (5-ASA) and azathioprine.

**Conclusion:** CASP1, SERPINA1, and CCL2 are autophagy-related hub genes of active UC. And SERPINA1 may serve as a new pharmacological autophagy regulator of UC, which provides a new target for the use of small molecules targeting autophagy in the treatment of active UC.

## Introduction

Ulcerative Colitis (UC) is a chronic and relapsing inflammatory bowel disease (IBD) (Park et al., 2014). Inflammatory lesions are commonly confined to the mucosal layer, begin in the rectum, and continuing to the proximal colon (Rubin et al., 2019; Matsuoka et al., 2018). The formation of UC is the result of the autoimmune and immune-mediated occurrence of genetically susceptible individuals. Immunity disorders, aberrant cytokine secretion, destruction of barrier function, and intestinal microbiota can lead to the occurrence of UC (Maloy and Powrie 2011).

Mammalian autophagy is a vital and fundamental cellular process for cells to adapt to the environment and maintain cell homeostasis (Morishita and Mizushima 2019; Liu and Sabatini 2020). This is an important self-protection mechanism. It continuously removes damaged organelles, abnormally folded proteins, or overproduced proteins (Retnakumar and Muller 2019). It is also effective in reducing the over-triggering of various self-defensive pathways such as inflammatory and immune responses (Kabat et al., 2016; Lee et al., 2016). The earliest study to identify the role of autophagy in intestinal homeostasis in IBD was the genome-wide association studies (GWASs) (Jostins et al., 2012). In Crohn’s disease (CD), another form of IBD, single nucleotide polymorphisms (SNPs) of genes that control autophagy and autophagy-dependent processes are one of the reasons for the increased susceptibility to CD (Larabi et al., 2020). Intestinal epithelial cells (IECs) play a crucial role in the intestinal barrier function. In addition to having nutrient absorption and metabolism, IECs create a mucosal barrier to protect the body from the invasion of exogenous factors including pathogenic bacteria. In a mouse model of chronic colitis, the selective deficiency of the autophagy gene ATG16L1 in IEC leads to a severe deterioration of the disease with increased secretion of pro-inflammatory cytokines and increased apoptosis of IEC cells (Pott et al., 2018). Studies have shown that the intestinal permeability of CD patients is increased, and this is related to the role of autophagy in intestinal tight junction barrier defects in IBD (Lerner and Matthias 2015; Nighot and Ma 2016; Citi 2018). Defects in autophagy could lead to disorders in the intestinal homeostasis, disturbances in the interactions between the intestinal microbiome and innate and adaptive immunity, and the reduction of the host’s ability to defend against intestinal pathogens (Mizushima 2018; Retnakumar and Muller 2019). Therefore, repairing the autophagy defect is expected to be a new treatment strategy for UC.

Studies have shown that the therapeutic effect of some UC drugs is partially mediated by regulating autophagy pathways (Hooper et al., 2017). The rapamycin analogues sirolimus and everolimus, whose pharmacological effects induce enhanced autophagy by inhibiting rapamycin complex 1 (mTORC1), be effective in improving intestinal symptoms in patients (Ke et al., 2016). For example, sirolimus and everolimus can significantly improve symptoms and intestinal healing in patients with severe refractory IBD (Massey et al., 2008; Dumortier et al., 2008). Sirolimus can induce clinical remission in 45% of refractory pediatric UC patients (Mutalib et al., 2014). Because of the vital role of autophagy in intestinal homeostasis and the influence of autophagy dysfunction in the pathogenesis of UC, it is of great therapeutic significance to find effective predictors of drug responsiveness. Therefore, this study aims to identify the critical autophagy-related regulators in the pathogenesis of UC through bioinformatics. We investigated the relationship between the autophagy-related regulators and the therapeutic effect of the current commonly used IBD drugs such as glucocorticoids, immunomodulators, aminosalicylates (5-ASAs), and biologics. This will help clarify the pharmacological mechanism and optimize the personalized therapy of active UC.

## Materials and Methods

### Datasets and Preprocessing

The gene expression profiles and corresponding clinical data were attained from the Gene Expression Omnibus (GEO) database (http://www.ncbi.nlm.nih.gov/geo/). A total of 232 autophagy-related genes were extracted from the Human Autophagy Database (HADb, http://www.autophagy.lu/index.html). The platform information corresponding to all data sets in the present study was shown in Table 1. Data analysis was performed with the R software (version 4.0.2). The “sva” R package was used to remove batch effects between different data sets, and then the merged data were normalized. The principal component analysis (PCA) was used to verify the results of batch correction.

**TABLE1:** The platform information corresponding to all data sets in the present study.

	Data set	Platform	Title
1	GSE53306	GPL14951	Illumina HumanHT-12 WG-DASL V4.0 R2 expression beadchip
2	GSE87466	GPL13158	[HT_HG-U133_Plus_PM] Affymetrix HT HG-U133 + PM Array Plate
3	GSE134025	GPL20115	Agilent-067406 Human CBC lncRNA + mRNA microarray V4.0
4	GSE107499	GPL15207	[PrimeView] Affymetrix Human Gene Expression Array
5	GSE59071	GPL6244	[HuGene-1_0-st] Affymetrix Human Gene 1.0 ST Array [transcript (gene) version]
6	GSE114527	GPL14951	Illumina HumanHT-12 WG-DASL V4.0 R2 expression beadchip
7	GSE92415	GPL13158	[HT_HG-U133_Plus_PM] Affymetrix HT HG-U133 + PM Array Plate
8	GSE38713	GPL570	[HG-U133_Plus_2] Affymetrix Human Genome U133 Plus 2.0 Array

### Evaluation of Immune Infiltration Patterns of Active UC

The relative abundances of 22 immune cells in the intestinal mucous tissue of active UC and healthy control were inferred by the “CIBERSORT” algorithm (Newman et al., 2015), which applies a matrix of reference gene expression values (LM22 file) to represent the minimum values of different cell types. The deconvolution algorithm was performed by analyzing 1,000 permutations and the LM22 file, which was downloaded from the CIBERSORT web portal (https://cibersort.stanford.edu/). The screening condition for qualified samples was *p* < 0.05. The results of the immune infiltration patterns were visualized using the “barplot” package, “corrplot” package, and “pheatmap” package in R.

### Differential Expression Analysis

Based on the original data, we utilized the “limma” R package to filter the differentially expressed genes (DEGs) between active UC samples and healthy samples, with adjusted *p*-value (adj. *p*) < 0.05 and |log2 fold change (FC)| > 1 as thresholds. The R packages “ggplot2” and “pheatmap” were used to visualize the results of differential expression analysis.

### Construction of Co-Expression Network and Identification of Modules

WGCNA, a systematic biological method that uses gene expression data to construct scale-free networks, can search gene modules with cooperative expression and identify correlations between modules and phenotypes (Langfelder and Horvath 2008). We constructed a co-expression network using the “WGCNA” R package. At first, we established a weighted adjacency matrix and the soft threshold power (β) was 13. Then the adjacency matrix was converted to a topological overlap matrix (TOM) to prepare for hierarchical clustering analysis (Ravasz et al., 2002). The gene modules were identified using a dynamic tree cut algorithm with a minimum size of 30, and gene modules were clustered according to the cutoff value of 0.4. Genes within an identified module had similar expression patterns and were strongly related to the occurrence of active UC.

### Functional Annotation and Pathway Enrichment Analysis

To further understand the underlying pathomechanism of active UC, the Gene Ontology (GO) and Kyoto Encyclopedia of Genes and Genomes (KEGG) analyses were performed using the “clusterProfiler” package based on the DEGs between healthy control and active UC.

### Statistical Analysis

The data in GSE114527, GSE92415, and GSE38713 were statistically analyzed with Graphpad Prism 8 (Graphpad Software Inc., United States). All data were represented as the mean ± SE for quantitative variables. One-way ANOVA was used for more than two comparison groups. All other statistical analysis was implemented by R (v4.1.0, http://www.R-project.org), and *p* < 0.05 was considered statistically significant.

## Results

### Data Preprocessing

**FIGURE 1:**
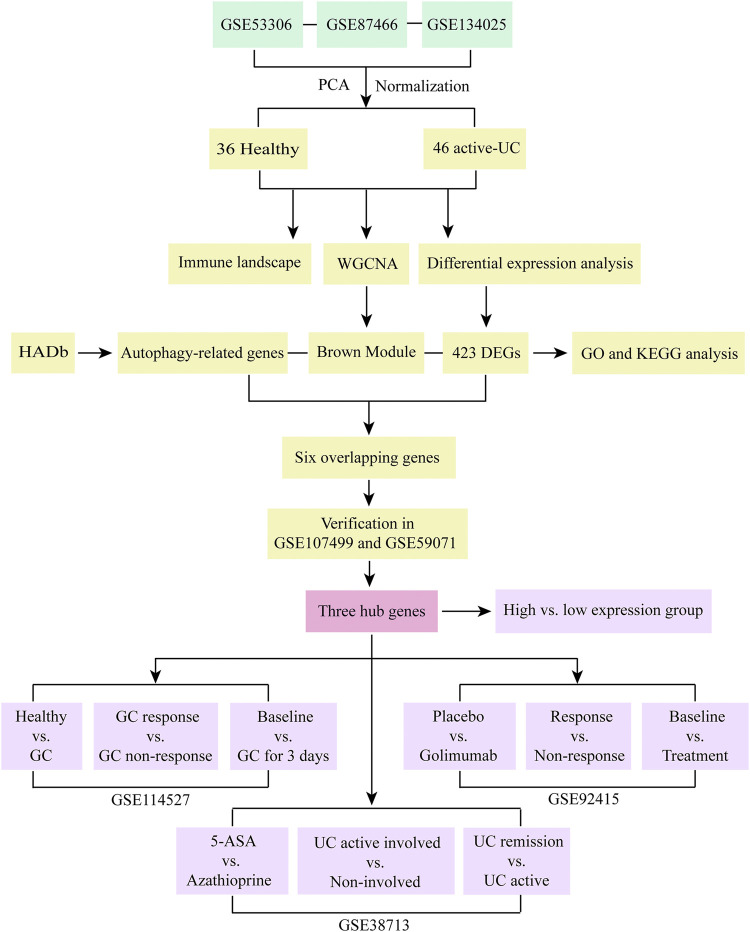
Research flow chart of this study. PCA, principal component analysis. UC, ulcerative colitis. WGCNA, weighted gene co-expression network analysis. HADb, the Human Autophagy Database. DEGs, differentially expressed genes. GO, the Gene Ontology. KEGG, the Kyoto Encyclopedia of Genes and Genomes analyses. GC, glucocorticoid. 5-ASA, 5-aminosalicylic acid.

**FIGURE 2:**
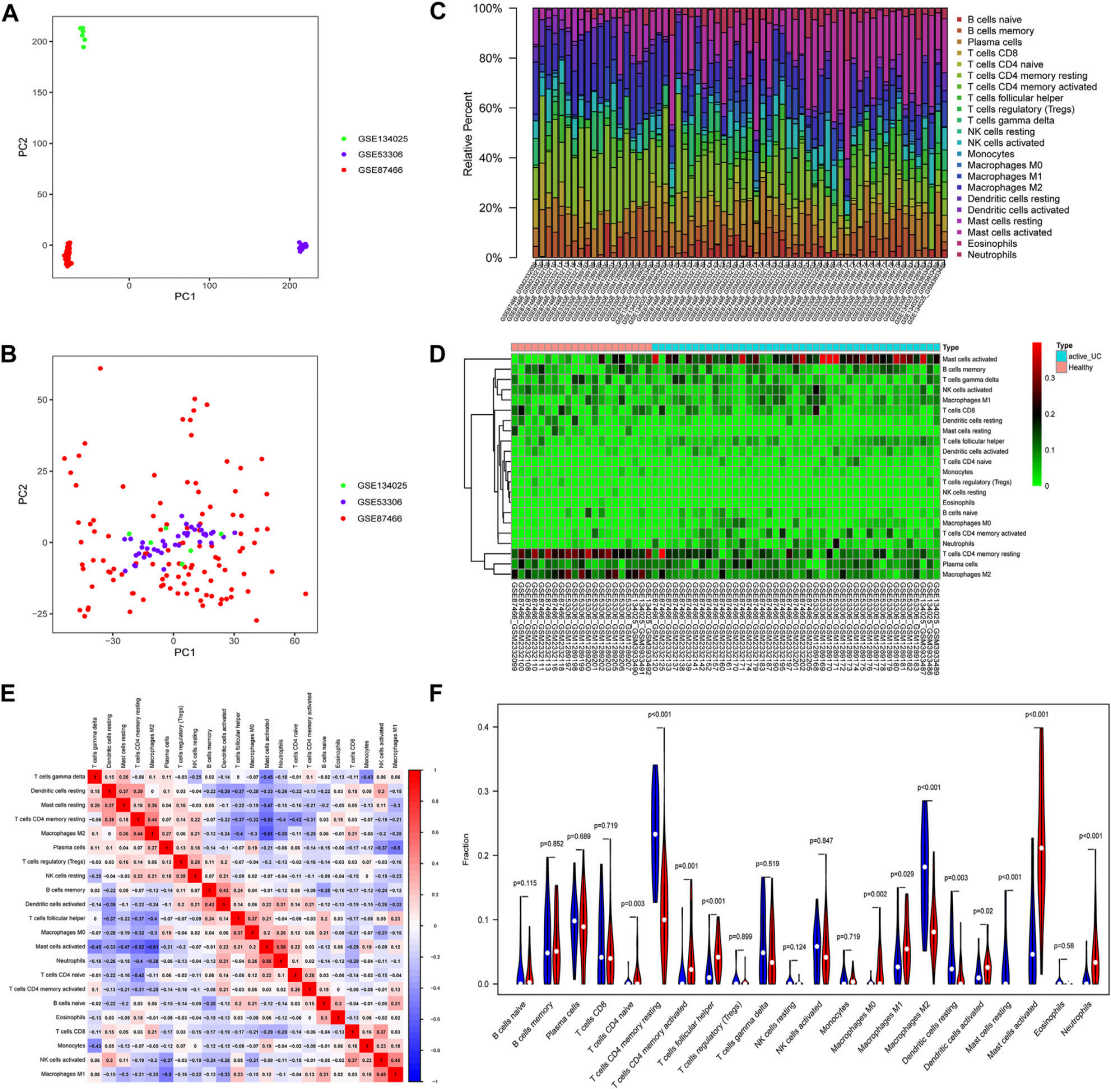
Principal component analysis (PCA) and immune-infiltrating landscape of active UC. The PCA of before **(A)** and after **(B)** batch correction of all samples. **(C)** The distribution of 22 immune cells in all active UC patients and healthy control samples in the total set. **(D)** The heat map delineated the abundance of the immune cell populations in each sample of active UC and healthy controls. **(E)** Correlation matrix of different infiltrating immune cells. The darker the blue, the stronger the negative correlation, and the darker the red, the stronger the positive correlation. **(F)** Comparison of immune cell infiltration in the intestinal mucosa between active UC patients and healthy control. Blue represents the healthy control group, and red represents the active UC group.

The analysis flow chart of this study was shown in Figure 1. The expression profile data of GSE53306, GSE87466, and GSE134025 downloaded from the GEO database were preprocessed and evaluated, and a total of 36 healthy controls and 46 active UC samples were obtained for subsequent analysis. The results of PCA revealed that the batch effect between three data sets was eliminated (Figures 2A,B).

### Immune-Infiltrating Landscape of Active UC

Various microbes, damaged organelles, and aggregates are some of the sources of inflammatory signals (Deretic et al., 2013; Deretic and Levine 2018). The cytoplasmic clearance of autophagy is crucial to the normative development, differentiation, and function of immune cells (Morishita and Mizushima 2019). UC is a chronic inflammatory intestinal barrier disease and mucosal immune dysregulation may be related to the pathogenesis of UC (Kobayashi et al., 2020). Therefore, to clarify the pathogenesis of active UC, we compared the difference of 22 immune cells infiltrating in intestinal mucosal of healthy controls and active UC patients through the CIBERSORT algorithm (Figures 2C–F). Filter out samples with *p* > 0.05, and finally obtain 43 active UC and 21 healthy controls from the total set. The histogram visualized the composition of the immune cell populations in each sample of active UC and healthy controls (Figure 2C). Figures 2D,F showed the abundance difference in immune cell infiltration of intestinal mucosal between active UC and healthy control. The immune cells with higher infiltration levels in the intestinal mucosa of patients with active UC than that of healthy controls include activated mast cells (*p* < 0.001), CD4^+^ naive T cell (*p* = 0.003), activated CD4^+^ memory T cells (*p* = 0.001), follicular helper T cells (*p* < 0.001), M0 macrophages (*p* = 0.002), M1 macrophages (*p* = 0.029), activated dendritic cells (*p* = 0.02), and neutrophils (*p* < 0.001). However, healthy controls were in possession of higher levels of infiltration of CD4^+^ memory resting T cells (*p* < 0.001), resting dendritic cells (*p* = 0.03), resting mast cells (*p* < 0.001), and M2 macrophages (*p* < 0.001) in intestinal mucosa. Besides, there was a weak correlation between the components of different immune cell infiltration (Figure 2E). Activated mast cells were slightly closely related to other immune cells. It had a weak negative correlation with M2 macrophages (r = −0.61) and CD4^+^ memory resting T cells (r = −0.52) and a weak positive correlation with neutrophils (r = 0.56).

### Identification of DEGs and Functional Annotation and Pathway Enrichment of DEGs

**FIGURE 3:**
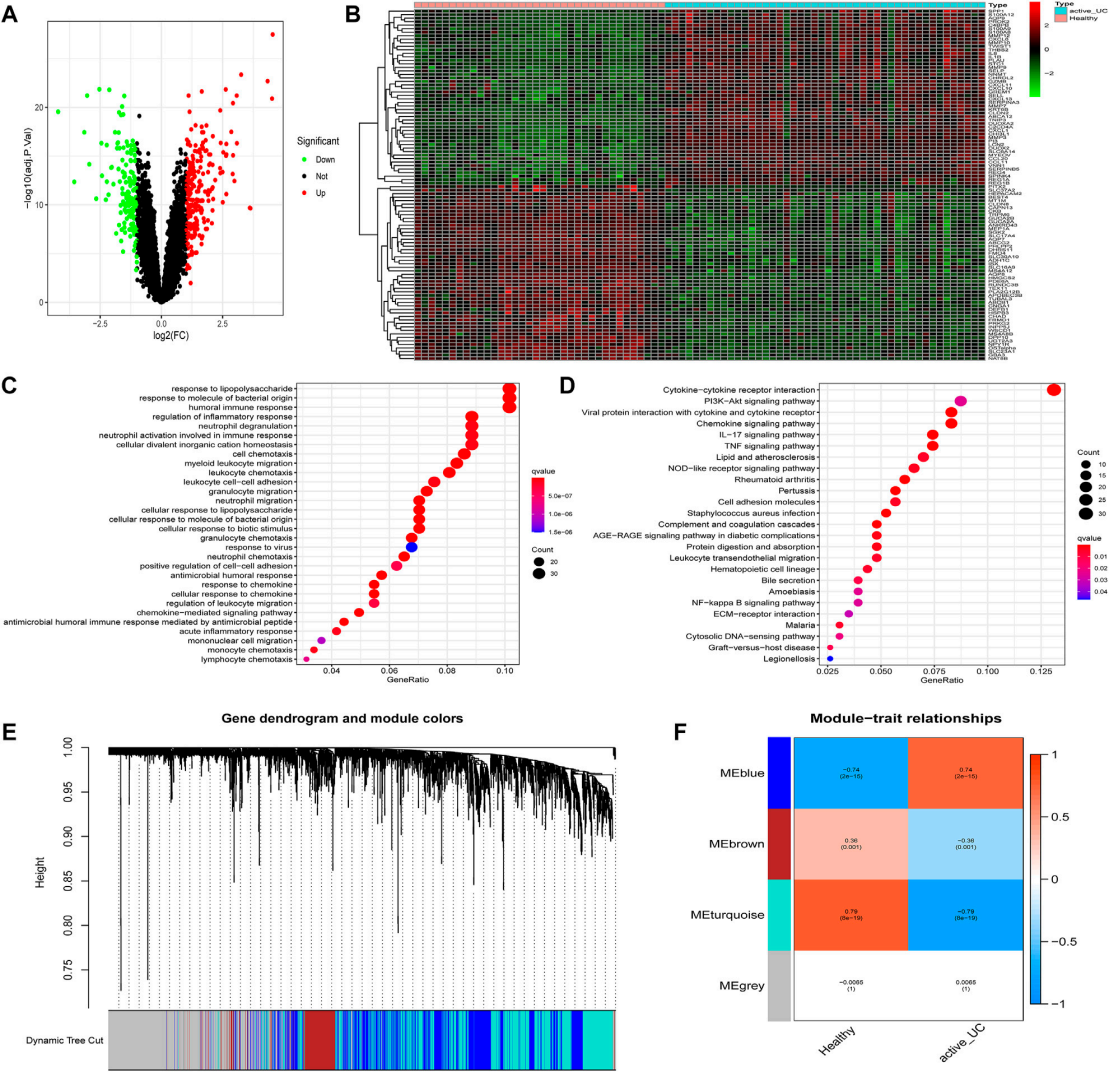
Identification of DEGs and modules associated with active UC. **(A)** Volcano map of DEGs in the intestinal mucosa between active UC patients and healthy control. Red represents up-regulated genes, green represents down-regulated genes, and black represents no significant difference genes. **(B)** The expression profiles of top 50 DEGs between active UC patients and healthy controls. The red to green colors represent the change from high to low expression. **(C)** GO enrichment analysis of DEGs. **(D)** KEGG pathway enrichment analysis of DEGs. **(E)** Module clustering dendrogram based on a dissimilarity measure (1-TOM). **(F)** Heatmap of the correlation between module eigengenes and active UC. DEG, differentially expressed genes. UC, ulcerative colitis. GO, the Gene Ontology. KEGG, the Kyoto Encyclopedia of Genes and Genomes analyses. TOM: topological overlap matrix.

Differential expression analysis was performed based on the expression profile of 36 healthy controls and 46 active UC patients from three different data sets (GSE53306, GSE87466, and GSE134025). We obtained a total of 423 DEGs, including 256 upregulated DEGs and 167 downregulated DEGs. The volcano plot and hierarchical clustering heatmap plot showed the distribution of DEGs between healthy controls and active UC (Figures 3A,B). The heatmap saw the expression pattern of the top 50 DEGs between active UC patients and healthy controls (Figure 3B).

Then, GO and KEGG enrichment analyses were performed on the 423 DEGs to get a better understanding of the potential molecular mechanisms and functions of active UC. Most of the GO terms of biological process (BP) were focused on the migration and chemotaxis of immune cells, such as neutrophils, myeloid leukocytes, and granulocytes (Figure 3C). Autophagy-mediated lipolysis was critical for neutrophil self-renewal by supplying free fatty acids to the mitochondrial respiration pathway necessary for neutrophil differentiation (Riffelmacher et al., 2017). Other significantly enriched GO terms were also compactly related to immune cell adhesion and immune response. Moreover, the enriched KEGG pathway showed in Figure 3D. A great majority of inflammation-related pathways were activated at the active UC group, including cytokine-cytokine receptor interaction, IL-17 signaling pathway, and TNF signaling pathway. More importantly, several pathways associated with autophagy were enriched, such as the PI3K-Akt signaling pathway and NF-kappa B signaling pathway. It was worth mentioning that the aberrant activation of inflammasome caused by autophagy-related protein deficiency was pivotal for the progression of inflammatory disease (Takahama et al., 2018).

### WGCNA Construction and Key Modules Identification

The WGCNA network could divide genes into different modules with similar biological functions (Bryan et al., 2018). We identified four modules in the module classification through WGCNA (Figure 3E). Based on the module eigengene (ME), and the turquoise (*r* = −0.79, *P* = 8e − 19) and blue (*r* = 0.74, *P* = 2e − 15) modules with higher connectivity were selected as featured modules (Figure 3F). This also indicated that the turquoise module was highly negatively correlated with the occurrence of active UC, while the blue module was strongly positively correlated with active UC. However, the other two modules had a correlation of less than 0.5 with active UC.

### Identification of Autophagy-Related Hub Genes

**FIGURE 4:**
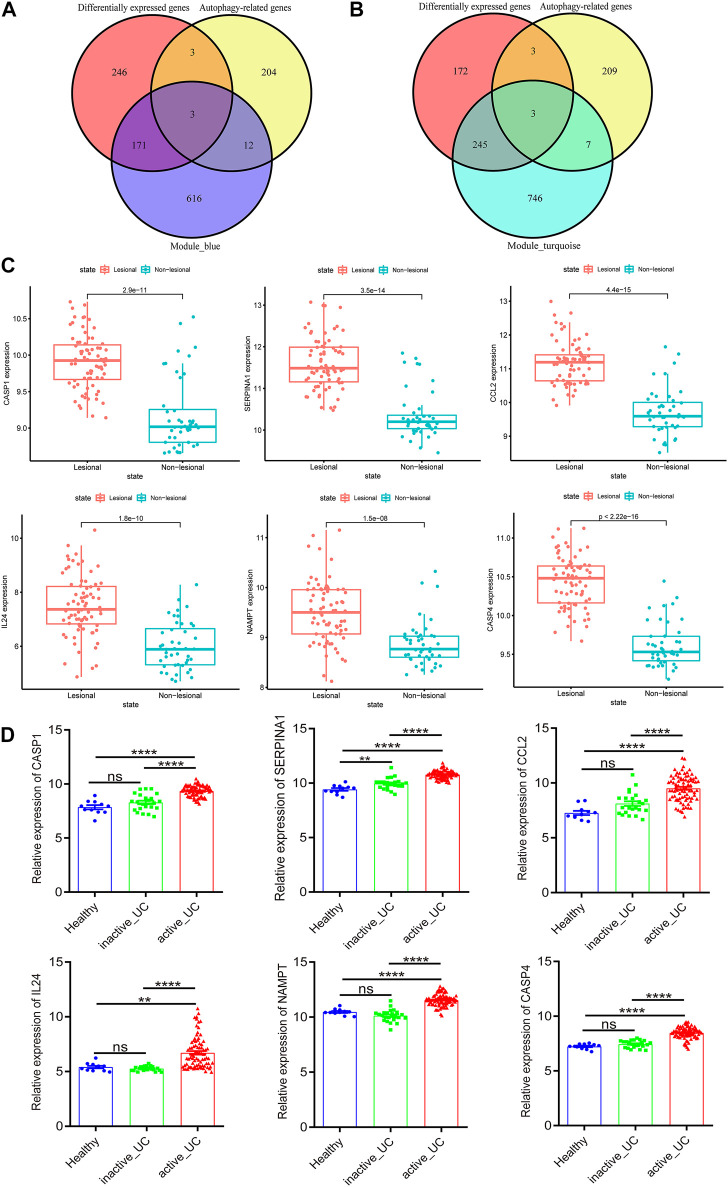
Identification and verification of autophagy-related hub genes. **(A)** Venn diagram shows the overlapping genes between differentially expressed genes, autophagy-related genes and the genes of blue module. **(B)** Venn diagram shows the overlapping genes between differentially expressed genes, autophagy-related genes and the genes of turquoise module. **(C)** The expression of CASP1, SERPINA1, CCL2, IL24, NAMPT, and CASP4 in the lesional and non-lesional intestinal mucosa of active UC patients in GSE107499. **(D)** The expression of CASP1, SERPINA1, CCL2, IL24, NAMPT, and CASP4 in the intestinal mucosa of healthy controls, inactive and active UC patients in GSE59071. UC, ulcerative colitis. *p < 0.05, **p < 0.01, ***p < 0.001, ****p < 0.0001, ns, not statistically significant.

To further narrow the range of autophagy-related hub genes, we used a Venn diagram to obtain the common genes of autophagy-related genes and DEGs with the genes in blue and turquoise modules, respectively (Figures 4A,B). Three hub genes were obtained from both two Venn analyses. Based on these results, we concluded that CASP1, SERPINA1, and CCL2 were autophagy-related hub genes with a remarkable positive correlation with active UC (Figure 4A), while IL24, NAMPT, and CASP4 were autophagy-related hub genes with a prominent negative correlation with active UC (Figure 4B).

### Verification of Autophagy-Related Hub Genes

Subsequently, we verified the differential expression of these six hub genes by another two data sets downloaded from GEO. GSE107499 contained an expression profile from inflamed and non-inflamed colon tissue from patients with active UC. Figure 4C revealed that CASP1 (*p* = 2.9e − 11), SERPINA1 (*p* = 3.5e − 14), CCL2 (*p* = 4.4e − 15), IL24 (*p* = 1.8e − 10), NAMPT (*p* = 1.5e − 08), and CASP4 (*p* = 2.22e − 16) were all upregulated in the lesion colon tissue of active UC patients. Furthermore, the expression of these six hub genes of active UC patients was higher than inactive UC patients and healthy controls in GSE59071 (Figure 4D). It was worth noting that, except for SERPINA1 (*p* < 0.01), there was no statistically significant difference in the expression of the other five hub genes in the intestinal mucosal tissues of healthy controls and inactive UC patients. Given the analysis results of WGCNA, the genes in the turquoise module were negatively correlated with active UC, and the genes in the blue module were positively correlated with active UC. Therefore, CASP1, SERPINA1, and CCL2 in the blue module were upregulated in the active UC samples, which was logical and could be used for further analysis.

**FIGURE 5:**
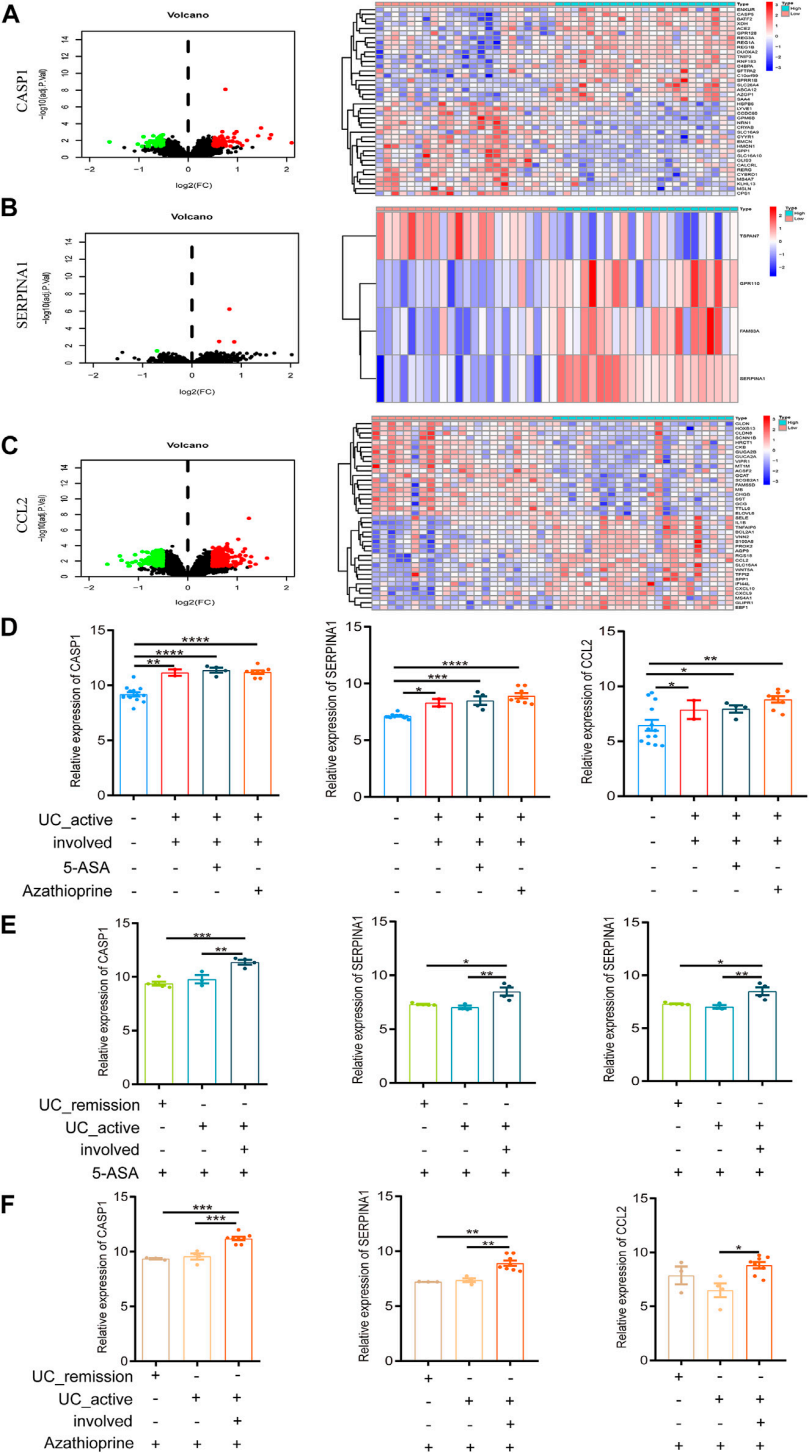
Intestinal mucosal invasion of active UC is associated with autophagy defects. **(A)** The Volcano map and expression profiles of top 20 DEGs between high and low CASP1 expression groups in active UC patients. **(B)** The Volcano map and expression profiles of top 4 DEGs between high and low SERPINA1 expression groups in active UC patients. **(C)** The Volcano map and expression profiles of top 20 DEGs between high and low CCL2 expression groups in active UC patients. **(D)** The relative expression levels of CASP1, SERPINA1, and CCL2 in the involved intestinal mucosa of active UC patients treated 5-ASA or azathioprine. **(E)** The relative expression levels of CASP1, SERPINA1 and CCL2 in involved and non-involved intestinal mucosa of active UC patients treated with 5-ASA. **(F)** The relative expression levels of CASP1, SERPINA1 and CCL2 in involved and non-involved intestinal mucosa of active UC patients treated with azathioprine. UC, ulcerative colitis. DEGs, differentially expressed genes. 5-ASA, 5-aminosalicylic acid. *p < 0.05, **p < 0.01, ***p < 0.001, ****p < 0.0001.

According to the median expression levels of CASP1, SERPINA1, and CCL2, 46 active UC patients in the comprehensive data set were divided into high and low expression groups. The heat map showed the top 20 genes with significant differences between the high and low expression groups based on the screening threshold as |log2FC| > 1 and *p* < 0.05. We could conclude that CASP1 (Figure 5A), SERPINA1 (Figure 5B), and CCL2 (Figure 5C) could divide active UC patients into two groups with significantly different expressions.

### Intestinal Mucosal Invasion of Active UC Is Associated With the Defect in Autophagy

GSE38713 was a data set containing whole-genome transcriptional analysis data of colonic biopsies from patients with active and inactive UC treated with 5-aminosalicylic acid (5-ASA) and azathioprine. It contained a total of 43 biopsies: 13 healthy controls, eight inactive UC, seven non-involved active UC, and 15 involved active UC. The inclusion and exclusion criteria for active and inactive UC patients were strict. We compared the expression of autophagy-related hub genes in the above samples. We found no difference in the expression of CASP1, SERPINA1, and CCL2 in involved intestinal mucosa of active UC patients after treatment with 5-ASA or azathioprine (*p* > 0.05, Figure 5D), which was still significantly higher than that of healthy controls. And the expression of CASP1, SERPINA1, and CCL2 showed no difference with or without drug treatment (*p* > 0.05, Figure 5D).

UC is a chronic relapsing gastrointestinal disease, and the alternation of remission and relapse is an important feature of it (Halmos and Gibson 2015). When treated with 5-ASA (Figure 5E) or azathioprine (Figure 5F), the expression of CASP1, SERPINA1, and CCL2 in the non-involved intestinal mucosa of active UC patients were not different from that of patients in remission, but significant differences were found between the involved and non-involved intestinal mucosa of active UC patients. In addition, after 5-ASA or azathioprine treatment, the expression of CASP1 and SERPINA1 in involved intestinal mucosa of active UC was significantly higher than that of UC patients in remission, but the expression of CCL2 was not different between the two groups (*p* > 0.05).

According to these results, we could speculate that the intestinal mucosal invasion of active UC patients may lead to the defect in autophagy, but not related to the treatment of 5-ASA or azathioprine. What’s more, no or less impaired autophagy may occur in the non-involved intestinal mucosa of active UC patients and remission patients.

### Glucocorticoid (GC) or Golimumab Responders Improve Impaired Autophagy of Intestinal Mucosal in Patients With Active UC by Reducing SERPINA1

**FIGURE 6:**
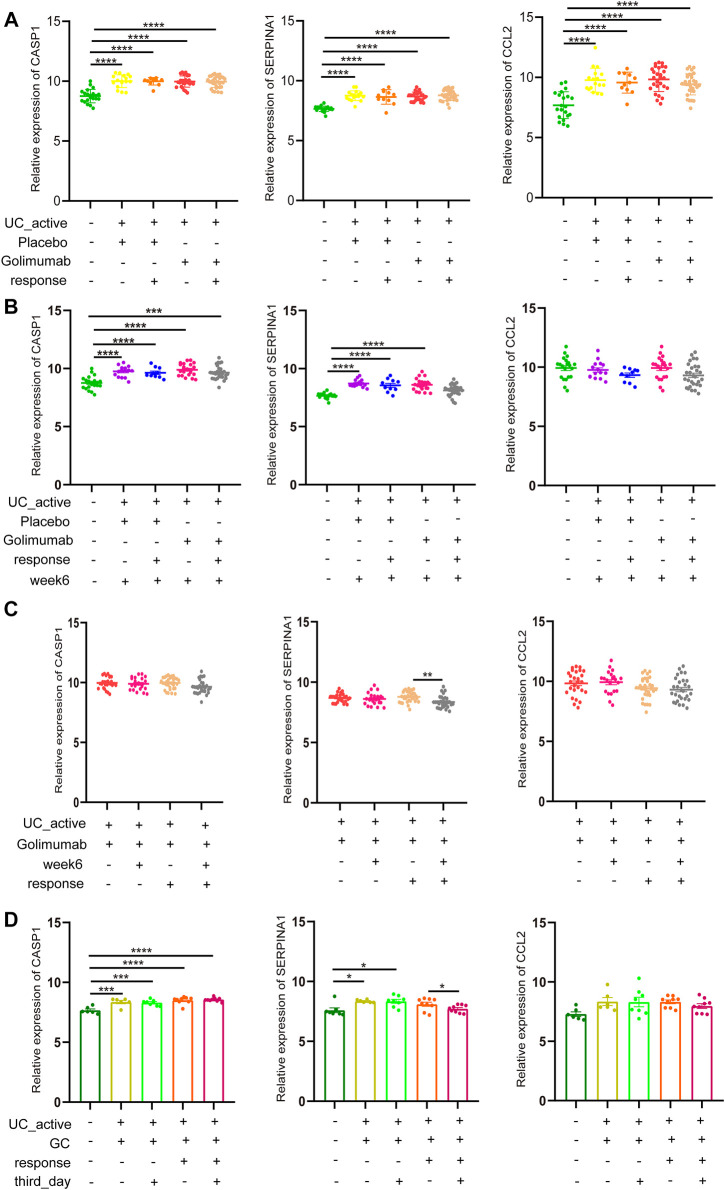
GC or golimumab responders improve impaired autophagy of intestinal mucosal in patients with active UC by reducing SERPINA1. **(A)** The relative expression levels of CASP1, SERPINA1, and CCL2 in the intestinal mucosa of active UC patients in responding and non-responding groups before golimumab treatment. **(B)** The relative expression levels of CASP1, SERPINA1, and CCL2 in the intestinal mucosa of active UC patients in responding and non-responding groups after 6 weeks of golimumab treatment. **(C)** The relative expression levels of CASP1, SERPINA1, and CCL2 in the intestinal mucosa of active UC patients in responding and non-responding groups before and after 6 weeks of golimumab treatment. **(D)** The relative expression levels of CASP1, SERPINA1, and CCL2 in the intestinal mucosa of active UC patients in responding and non-responding groups before and after GC treatment for 3 days. UC, ulcerative colitis. GC, glucocorticoid. *p < 0.05, **p < 0.01, ***p < 0.001, ****p < 0.0001.

Golimumab is a fully human IgG1 Kappa monoclonal antibody against tumor necrosis factor (TNF)-α (Sandborn et al., 2014). Subcutaneous injections of golimumab can induce a clinical response, remission, and mucosal healing, and improve quality of life in patients with active UC. It has been used in clinical practice for many years in adult patients with UC. In GSE92415, the expression of CASP1, SERPINA1, and CCL2 in the intestinal mucosa of active UC patients in the golimumab response and non-response groups was not different compared to the placebo group before golimumab treatment, which was higher than that in the healthy control group (Figure 6A). However, after 6 weeks of golimumab treatment, the expressions of CASP1 were still significantly higher than those of healthy controls (*p* < 0.0001, Figure 6B). It is worthwhile mentioning that the expression of SERPINA1 in the golimumab response group was significantly reduced (*p* < 0.05). The expression of CCL2 was dramatically reduced, and there was no statistical difference with the healthy control group (*p* > 0.05). While the placebo group showed the same changes. Therefore, after the treatment of golimumab, whether CCL2 was involved in the autophagy repair of impaired intestinal mucosal in active UC patients remained to be further studied. Besides, the expression of CASP1 and CCL2 in the intestinal mucosa of active UC patients with or without golimumab treatment for 6 weeks in the golimumab response group did not change. And the same results were also observed in the non-responsive groups. It is important to highlight that the expression of SERPINA1 was significantly down-regulated in the golimumab response group after receiving golimumab treatment for 6 weeks (Figure 6C).

GC can prevent TNF-induced increase intestinal permeability and fatal shock, and also plays an essential role in the treatment of active UC (Ballegeer et al., 2018). In GSE114527, there was no difference in the expression of CASP1 in the intestinal mucosa of active UC patients in the GC-responsive and non-responsive groups (Figure 6C). After 3 days of GC treatment, there was little change in CASP1 expression. For SERPINA1, after the treatment of GC for 3 days, there was no difference between the GC response group and the healthy control. In the GC non-responsive group, SERPINA1 expression in the intestinal mucosa of active UC patients was not different before and after GC treatment for 3 days, which was always higher than that of healthy controls (*p* < 0.05). These results indicated that the response to GC therapy could improve the autophagy damage of intestinal mucosa of active UC patients through SERPINA1 involved pathways. It must also be mentioned that CCL2 did not differ across all groups (*p* > 0.05).

Based on our findings, it can be concluded that among GC or golimumab responders, repair of impaired autophagy of intestinal mucosal in patients with active UC depends on SERPINA1.

## Discussion

The role of autophagy defects in disrupting intestinal homeostasis, affecting the composition of gut microbes, impairing intracellular bacterial clearance, and exacerbating intestinal inflammation is becoming more and more noteworthy (Larabi et al., 2020). The development of new therapies for the treatment of active UC based on the regulation of autophagy will be more promising. In this study, we identified three autophagy-related genes that were highly positively correlated with active UC. More importantly, CASP1, SERPINA1, and CCL2 may serve as new pharmacological autophagy regulators of UC, which provides new targets for the use of small molecules targeting autophagy in the treatment of active UC. These findings reveal a crucial new strategy for the employment of small molecules targeting autophagy in the treatment of active UC.

Multiple studies have shown that CASP1 plays an essential role in promoting inflammatory responses and autophagy defects. The myotubularin-related protein 3 (MTMR3) reduced the level of autophagy induced by pattern recognition receptor (PRR) while increasing PRR-induced CASP1 activation, IL-1β secretion, the activation of the NFκB signaling, and finally increased the secretion of overall cytokines in IBD (Lahiri, et al., 2015). In oral lichen planus (OLP) tissue, the expression of inflammatory factors and immune-related genes was positively correlated with the expression of CASP1 (Zeng, et al., 2021). As we all know, the normal execution of the lysosomal function is critical for the orderly maintenance of the autophagy process. CASP1 is one of the components of inflammasome (Takahama et al., 2018). In macrophages, the release of the lysosomal protease CTSB (cathepsin B) led to lysosomal damage and inhibition of autophagy, which was sensed by the NLRP3 (NLR family, pyrin domain containing 3) inflammasome and activated the NLRP3-CASP1 pathway (Chiu et al., 2016; Halle et al., 2008). This would lead to the processing and release of IL-1α, IL-1β, and IL-18, which induce inflammation and adaptive immune responses. CASP1 was involved in impaired autophagy leading to abnormal activation of the inflammasome, and this was a vital cause of inflammatory diseases such as active UC.

Soendergaard et al. identified SERPINA1 as a potential serum biomarker to identify mild or moderate disease activity in UC (Soendergaard et al., 2015). Besides, SERPINA1 plays a role in promoting human susceptibility to IBD (Kotlowski et al., 2008). SERPINA1 was a participant in the progression of colorectal adenocarcinoma (Zhang et al., 2021). CCL2 belongs to a superfamily of secreted proteins involved in immune and inflammatory processes. Signaling by binding and activating CCR2 induces a strong chemotactic response and mobilization of intracellular calcium ions (Paavola et al., 1998), showing strong chemotactic activity to monocytes and basophils (Weber et al., 1996). The expression of CCL2 was increased in LPS-treated CT-26 (mouse colon cancer cell) (Shi et al., 2019), which was consistent with our study that CCL2 expression was up-regulated in inflammatory states. Tumor necrosis factor (TNF), produced by macrophages, monocytes, and activated T cells, is a pivotal inflammatory mediator in the progression of UC (Papadakis and Targan 2000). As our analysis shows, CD4^+^ naive T cell, activated CD4^+^ memory T cells, follicular helper T cells, M0 macrophages, and M1 macrophages showed increased infiltration in patients with active UC (Figure 2F), suggesting that the expression of TNF was also elevated in patients with active UC, which would promote the vital role of anti-TNFa biological agents in anti-IBD intestinal inflammation. Infliximab plays a role in the treatment of UC by indirectly inducing T cell apoptosis by acting on transmembrane TNF expressed by CD14^+^ macrophages (Atreya et al., 2011). In UC patients treated with infliximab for 14 weeks, CCL2 expression in serum and intestinal tissue of responders was lower than that of non-responders (Magnusson et al., 2015). In our study, the responders of golimumab showed no difference in CCL2 expression in the intestinal mucosa after treatment compared with non-responders. This may indicate a difference in the mechanism of therapy between golimumab and infliximab for UC. Magnusson et al. suggested that the reduction of serum CCL2 levels in UC patients following infliximab treatment was strongly associated with monocyte activation (Magnusson et al., 2015).

In this study, the genes (IL24, NAMPT, and CASP4) in the turquoise module were not further analyzed. Compared with the control group, IL24 expression was increased in serum and colon samples from pediatric IBD patients and dextran sulfate sodium (DSS) induced colitis mouse models (Onody et al., 2021). Besides, NAMPT was intensively upregulated in inflammation, including IBD (Gerner et al., 2018). FK866, a small molecule inhibitor of NAMPT, improved colitis induced by DSS and inhibited the tumorigenesis of inflammation-related tumors. The expression of CASP4 was elevated in biopsies of UC patients with severe inflammation (Flood et al., 2015). However, our WGCNA analysis showed that the genes in the turquoise module were negatively correlated with active UC, contradicting the view that these genes were dramatically up-regulated in UC patients.

Autophagy inhibits the activation of the innate immune response (White et al., 2021). As shown in Figures 2C–F, compared with the normal control group, the active UC group showed the activation of various types of immune cells, which also indicated the presence of autophagy defects in the intestinal mucosa of active UC patients. Analysis of the biological process (BP) GO terms of DEGs revealed that DEGs were mainly enriched in humoral immune response and regulation of inflammatory response. Among them, more DEGs were enriched in the chemotaxis, activation, adhesion, and migration of immune cells, which include neutrophils, leukocytes, and myeloid leukocytes. In our study, the weak correlation between different immune cell infiltration components may be due to the high heterogeneity of immune cell infiltration levels in each sample. The cause of inducing inflammation in defective autophagy may be due to the failure of clearance of damaged proteins, organelles, and bacteria. Mice lacking essential autophagy genes showed increased secretion of pro-inflammatory cytokines such as interferons (IFNs), tumor necrosis factor α (TNFα), CCL2, and C-X-C motif chemokine ligand 10 (CXCL10) (Karsli-Uzunbas et al., 2014). Studies on autophagy in tumors suggest that the inactivation of autophagy in tumor cells or hosts promotes the production of cytokines and activates T cells to adequately kill tumors. But it also leads to an enormous depletion of T cells, which is ultimately beneficial for tumor growth (White et al., 2021).

The pharmacological effects of 5-ASA in the treatment of IBD involve inhibition of leukocyte motility, interference of TNFα, TGF- β, IL-1, and NFκB, and promotion of intestinal epithelial cells to maintain normal redox level and self-renewal (Campregher and Gasche 2011). It is commonly used to induce and maintain remission in patients with mild to moderate UC (Kyrmizi et al., 2013). Zhou et al. found that an autophagy-related pathway was activated in intestinal mucosal tissues of DSS-induced C57BL/6 mice after 5-ASA treatment (Zhou et al., 2021). In our study, autophagy-related genes such as CASP1, SERPINA1, and CCL2 did not show significant changes in the intestinal mucosa of UC patients treated with 5-ASA compared with the untreated group, which may be because these three genes were not in the autophagy pathway discovered by Zhou et al. (2021), or due to the species differences between mice and humans. Azathioprine, an immunosuppressant, can maintain remission in moderate-to-severe patients, albeit works slowly (Guijarro et al., 2012). Protective autophagy occurred in hepatocytes during thiopurines treatment (Guijarro et al., 2012), which suggested that the combination therapy of thiopurines and autophagy-inducing drugs could reduce their adverse reactions and improve their efficacy and safety. Corticosteroid neutralizes the immune system or blocks the pro-inflammatory responses by inhibiting transcription of genes associated with pro-inflammatory cytokines such as IL-1, IL-6, and TNFα and reducing the stability of their messenger RNA (Danese and Peyrin-Biroulet 2013). What’s more, the corticosteroid is the first-line therapy for active UC. Studies have shown that the clinical response of UC patients to GC was related to mTORC1. After 3 days of corticosteroid treatment, DNA damn-induced transcript 4 (DDIT4), an inhibitor of mTORC1 activity, was upregulated in respondents (Hooper et al., 2017). Golimumab is a monoclonal antibody against TNFa, which is known to be involved in autophagy regulation in a variety of cells, such as synovial fibroblasts of patients with rheumatoid arthritis (Connor et al., 2012) and atherosclerotic vascular smooth cells (Jia et al., 2006). It had been shown that TNF-α led to sarcomeric dysfunction and remodeling by causing autophagy defects and enhancing myofibril degradation in congestive heart failure (Opperman and Sishi 2015). In our study, the expression of autophagy-related gene SERPINA1 was significantly down-regulated in UC patients who responded to golimumab after 6 weeks of treatment (Figure 6C). Based on these findings, it can be concluded that anti-TNFα therapy may activate autophagy in UC.

## Conclusion

In conclusion, we identified that CASP1, SERPINA1, and CCL2 are autophagy-related hub genes of active UC, and SERPINA1 has powerful potential as a new pharmacological autophagy regulator of active UC. Our study provides new targets for small molecules targeting autophagy to treat active UC and contributes to understanding the autophagy-related molecular mechanisms involved in the occurrence of active UC.

## Data Availability

The original contributions presented in the study are included in the article/supplementary material, further inquiries can be directed to the corresponding authors.
